# The Influence of Baseline Alzheimer's Disease Severity on Cognitive Decline and CSF Biomarkers in the NILVAD Trial

**DOI:** 10.3389/fneur.2020.00149

**Published:** 2020-03-06

**Authors:** Laila Abdullah, Fiona Crawford, Magda Tsolaki, Anne Börjesson-Hanson, Marcel Olde Rikkert, Florence Pasquier, Anders Wallin, Sean Kennelly, Ghania Ait-Ghezala, Daniel Paris, Suzanne Hendrix, Kaj Blennow, Brian Lawlor, Michael Mullan

**Affiliations:** ^1^Roskamp Institute, Sarasota, FL, United States; ^2^Archer Pharmaceuticals, Sarasota, FL, United States; ^3^Department of Neurology, Aristotle University of Thessaloniki, Thessaloniki, Greece; ^4^Department of Psychiatry and Neurochemistry, Institute of Neuroscience and Physiology, Sahlgrenska Academy, University of Gothenburg, Gothenburg, Sweden; ^5^Department of Geriatric Medicine, Radboudumc Alzheimer Center, Donders Institute of Medical Neurosciences, Radboudumc, Nijmegen, Netherlands; ^6^CHU Lille, Univ. Lille, DISTALZ Laboratory of Excellence, Lille, France; ^7^Department of Psychiatry and Neurochemistry, Institute of Neuroscience and Physiology, The Sahlgrenska Academy at University of Gothenburg, Gothenburg, Sweden; ^8^Trinity College Dublin, College Green, Dublin, Ireland; ^9^Department of Age Related Healthcare, Tallaght Hospital, Dublin, Ireland; ^10^Pentara Corporation, Millcreek, UT, United States; ^11^Clincial Neurochemistry Laboratory, Sahlgrenska University Hospital/Mölndal, Göteborg, Sweden

**Keywords:** mild Alzheimer's disease, nilvadipine, exploratory analysis, cognitive decline, cerebrospinal fluid Aβ42/Aβ40 ratios

## Abstract

We examined the effects of a dihydropyridine calcium channel blocker nilvadipine with anti-inflammatory properties on cognition and cerebrospinal fluid (CSF) biomarkers by baseline Alzheimer's disease (AD) severity. Exploratory analyses were performed on the dataset (*n* = 497) of a phase III randomized placebo-controlled trial to examine the response to nilvadipine in AD subjects stratified by baseline AD severity into very mild (MMSE ≥ 25), mild (MMSE 20-24) and moderate AD (MMSE < 20). The outcome measures included total and subscale scores of the Alzheimer's Disease Assessment Scale Cognitive 12 (ADAS-Cog 12), the Clinical Dementia Rating Scale sum of boxes (CDR-sb) and the AD composite score (ADCOMS). Cerebrospinal fluid biomarkers Aβ38, Aβ40, Aβ42, neurofilament light chain (NFL), neurogranin, YKL-40, total tau and P181 tau (ptau) were measured in a subset of samples (*n* = 55). Regression analyses were adjusted for confounders to specifically examine the influence of nilvadipine and baseline AD severity on cognitive outcomes over 78-weeks. Compared to their respective placebo-controls, nilvadipine-treated, very mild AD subjects showed less decline, whereas moderate AD subjects showed a greater cognitive decline on the ADAS-Cog 12 test and the ADCOMS. A lower decline was observed after nilvadipine treatment for a composite memory trait in very mild AD subjects and a composite language trait in mild AD subjects. Cerebrospinal fluid Aβ42/Aβ40 ratios were increased in mild AD and decreased in moderate AD patients treated with nilvadipine, compared to their respective controls. Among moderate AD subjects, levels of ptau, total tau, neurogranin and YKL-40 increased in subjects treated with nilvadipine compared to placebo. These studies suggest that baseline AD severity influenced the treatment outcome in the NILVAD trial and that future clinical trials of nilvadipine should be restricted to mild and very mild AD patients.

**Trial Registration:** NCT02017340 Registered 20 December 2013, https://clinicaltrials.gov/ct2/show/NCT02017340

EUDRACT Reference Number 2012-002764-27 Registered 04 February 2013, https://www.clinicaltrialsregister.eu/ctr-search/search?query=2012-002764-27

## Introduction

Alzheimer's disease (AD) is the most common neurodegenerative disease, affecting nearly 5.3 million US citizens. By 2050, the prevalence of AD is expected to reach 13 million in the United States alone and 100 million worldwide. The presence of amyloid plaques and neurofibrillary tangles in the brain are key hallmarks of AD (1–3) and are also accompanied by cerebrovascular disease, α-synuclein and TDP-43 deposits and inflammation (4–6). Recent clinical trials have shown that moderate AD patients, with established brain amyloid and tau pathologies, do not benefit cognitively from current therapeutic approaches, although some trials have shown potential benefits in mild and early stage AD patients (7–11). Therefore, there is a general consensus that early and mild AD patient populations may be more appropriate for a number of potential therapeutic approaches.

Nilvadipine is a dihydropyridine (DHP) calcium channel blocker currently approved in Europe and Asia as an anti-hypertensive drug. In preclinical studies, we have previously shown that nilvadipine has anti-inflammatory properties that are due to its ability to inhibit spleen tyrosine kinase (Syk), which results in increased Aβ clearance from the brain, lowered Aβ production and reduced tau hyperphosphorylation and inflammation (12–16). As such, nilvadipine may represent a novel, multimodal, disease-modifying therapy for AD. A small clinical trial of nilvadipine in mild cognitive impairment (MCI) patients showed reduced conversion to AD in the subjects treated with nilvadipine compared to those on amlodipine, which, in contrast to nilvadipine, does not penetrate the blood brain barrier (BBB) (17). A phase III multi-center, double-blinded, randomized, placebo-controlled clinical trial was conducted in Europe to test the efficacy of nilvadipine in treating AD (the NILVAD trial). In the NILVAD trial, when analyzed as a single population, combined mild and moderate AD subjects did not benefit from nilvadipine treatment. However, preplanned subgroup analyses indicated that, compared to placebo-treated controls, nilvadipine-treated mild AD subjects (baseline MMSE ≥ 20) showed cognitive benefits whereas moderate AD subjects (baseline MMSE < 20) showed worsening of cognition (18). These findings support further exploration of the treatment effects of nilvadipine in AD patients based on the disease severity at baseline.

The NILVAD cohort was composed of both mild and moderate AD groups which, in other studies (depending on their disease stage at baseline), experience differential cognitive decline (19). These differences in rates (and type) of cognitive decline reflect the sequential spreading of amyloid pathology in different brain regions. For instance, neuronal loss in early AD starts within the medial temporal lobe (MTL), which is primarily involved with memory function. With the advancement of AD, further degeneration occurs within the parietal, frontal, and occipital lobes, which involve language processing and praxis (19). We therefore performed unplanned exploratory analyses of data from the NILVAD trial (18) by further stratifying the study population by baseline AD severity. We also evaluated memory, language and praxis domains by grouping the AD Assessment Scale-Cognitive (ADAS-Cog) 12 subscales, as previously defined by 19. Additionally, we calculated a modified AD Composite Score (ADCOMS) using subscales from the ADAS-Cog 12 and the Clinical Dementia Rating Scale Sum of Boxes (CDR-sb) since such an approach has better sensitivity in detecting cognitive decline in mild AD compared to either test alone (20). In a subset of the study population stratified by AD severity at baseline, we examined cerebrospinal fluid (CSF) biomarkers, including Aβ, phosphorylated tau (ptau), total tau, YKL-40, neurogranin and neurofilament light chain (NFL). We anticipated that these exploratory analyses would help understand the impact of AD severity at baseline on differential response to nilvadipine over the course of an 18-month clinical trial.

## Methods

### Study Design and Participants

This 18-month phase III double-blind, placebo-controlled, randomized clinical trial was conducted in 9 countries across Europe [see elsewhere for additional details; (21)] and funded by the European Commission under a Framework 7 Programme Health Theme collaborative project grant. A separate Scientific Advisory Board, an independent Ethics Advisory Board and an independent Data Safety Monitoring Board were involved in the oversight of the trial. The study protocol and associated documents were approved by Research Ethnics Committee and Institutional Review Boards (IRB) for all study sites (see the ethics statement below for the full list of IRB by each country). A written consent for trial participation was obtained following a full explanation of the risks and benefits of the trial to potential participants [see elsewhere for details on the study leaflets; (21)]. A written consent was obtained from patients who had the ability to provide a consent as well as from the caregivers at the screening visit prior to initiating the study process. The procedure for obtaining informed consent from a participant with reduced decision-making capacity was conducted in accordance to the national laws of each country and assessed by the relevant bodies in each country. The sample size calculations for the main trial were based on the mean difference of 3.5 and standard deviation (SD) of 9 between the treated and control groups and as previously described elsewhere (21). The block randomization was performed using an online system hosted by the Clinical Trial Unit at the King's College London. Blocks of varying sizes were used. The randomization was at the subject level and stratified by country site, see elsewhere for more details (21). All study investigators and patients were blinded to the treatment assignment. There were no interim analyses in this trial.

The full details of the inclusion and the exclusion criteria are provided elsewhere (18, 21). Briefly, inclusion criteria for the study required that participants should be over the age of 50 and have a diagnosis of mild or moderate probable AD according to the established guidelines from the National Institute of Neurological and Communicative Disorders and Stroke/Alzheimer's Disease and Related Disorders Association Inc. (NINCDS-ADRDA) and the Alzheimer's Association, and having a baseline Mini-Mental State Examination (MMSE) score of ≥12 and ≤27 (21). A total of 569 subjects were screened for eligibility and 511 were randomized into the trial with 258 were assigned to the placebo group and 253 assigned to the nilvadipine group, of which one dropped out due to blood pressure measurements being out of range, leaving 252 in this group. Of these 510 subjects, 11 were lost to follow-up and 2 withdrew consent, leaving 497 subjects in the modified intention-to-treat (mITT) dataset, see 18 for additional details. Data from subjects in the mITT set were used for these additional exploratory analyses below. At baseline, each subject was randomly assigned to either 8 mg of Nilvadipine or placebo once a day, and each study subject was required to take the capsule orally after breakfast for 78 weeks. The primary outcome measures were the 12-item Alzheimer's Disease Assessment Scale–cognitive sub-scale 12 (ADAS-Cog 12) and the Clinical Dementia Rating scale sum of boxes (CDR-sb), and these tests were administered at four time-points (baseline, and weeks 13, 52, and 78).

### Cerebrospinal Fluid Biomarker Measurements

Cerebrospinal fluid samples were available for 94 subjects at baseline but both before and after treatment were available for 55 subjects. Collection of CSF collection was performed using a standardized sub-study protocol described elsewhere (22). Briefly, CSF collection by lumbar puncture was carried out between the screening and the baseline visit (within the 21-day window) and at the treatment termination (between 78 and 82 weeks). The lumbar puncture was performed using routine antiseptic cleansing and anesthesia with the patient in a reclining/sitting position. The lumbar puncture was performed using a Spinal Needle Quincke Type Point 0.7 × 75 mm (75–90 mm) that was inserted between L3/L4 or L4/L5 interspaces. Approximately 10 ml of CSF was collected in 15 ml polypropylene tubes. After gentle mixing, samples were centrifuged at 2,000 × g for 10 min at 4°C to remove cells and debris, and then 1ml aliquots were prepared using polypropylene cryovials and subsequently frozen at −80°C until further use. Levels of Aβ38, Aβ40, and Aβ42 were quantified using the Meso-scale discovery (MSD) platform as previously described (23). Levels of total tau and ptau (P181) were measured using commercially available sandwich ELISA kits (INNOTEST; Fujirebio) as per the manufacturer's instructions and as previously described (23). All analyses were performed by board-certified laboratory technicians blinded to clinical information. We applied CSF biomarker criteria using total tau (>350 pg/ml) and P181 tau (>60 pg/ml) cut-offs as defined by (24). An Aβ42/Aβ40 ratio cut-off of <0.82 was used based on the concordance figures with amyloid PET imaging (Blennow, unpublished data) to compare baseline values with the clinical AD diagnosis. In this subsample, approximately 91% of clinically diagnosed AD subjects also met CSF biomarker criteria for AD. This subset was representative of the whole study population with respect to sex, AD severity and APOE ε4 carrier status. Additional CSF biomarker measurements included YKL-40, NFL and neurogranin and these analyses were performed as previously described elsewhere (25–27).

### APOE Genotyping

Apolipoprotein E genotypes were available on a subset of subjects (*n* = 328). The Gentra Puregene Kit (Gentra Systems) was used to purify DNA from frozen whole blood according to the manufacturer's instructions and as previously described. The EzWay Direct APOE Genotyping Kit, (Koma Biotechnology), was used in accordance with the manufacturer's instructions; specifically amplified DNA fragments corresponding to different APOE alleles were separated by electrophoresis in a Ethidium Bromide stained 2% metaphor and 1% agarose gel. All genotypes were then verified using rapid PCR with high-resolution melting analysis according to the manufacturer's instructions (Novallele Genotyping).

### Statistical Analyses

General demographic characteristics across AD subgroups by treatment within the modified intent to treat (mITT) dataset were compared using either ANOVA or the Chi-square test, as applicable. Mixed linear model (MLM) regression was used to examine the main effects and the interactions between treatment, time (time points of study visits at 13, 52, and 78 weeks) and AD severity at baseline. As we were interested in the independent contributions of the baseline AD severity and treatment effect over time on the cognitive outcomes, these analyses were also adjusted to account for the confounding effects of gender and ε4 carrier status (coded as those with the presence of ε4, without ε4 and those with no genotype information since not all mITT subjects had APOE genotypes available) and the confounding effects of age at which subjects left education (referred to as “education” hereon). To account for the treatment effect modification observed in the subgroup analyses, this model also included interactions between time and APOE; treatment and APOE; time, treatment and APOE; time and gender; treatment and gender; and time, treatment and gender. Interactive terms were also included for education and time and for treatment and education to account for education imbalance across AD severity subgroups. All of these variables were considered fixed factors. Subjects and country were treated as random factors. The autoregressive covariance structure was used in these MLM analyses. The outcome variables included change in the total ADAS-Cog 12 scores and change in composite scores from the ADAS-Cog 12 for different cognitive traits, CDR-sb and the ADCOMS.

We also applied Principal Component Analysis (PCA) to minimize multicollinearity and achieve dimension-reduction for data on sub-scales from the ADAS-Cog 12 and CDR-sb for all visits. This method was used as an unsupervised procedure for achieving data-reduction and for identifying treatment responses in subgroups of subjects based on their baseline AD severity. The Kaiser-Meyer-Olkin (KMO) measure of sampling adequacy and Bartlett's test for sphericity were used to ensure adequacy for PCA analysis (KMO value of > 0.6 and Bartlett *p*-value < 0.05). Variables with eigenvalues of ≥1 were retained and PCA components (Factors) were extracted using varimax with Kaiser normalization for rotation in order to simplify and clarify the data structure. Individual ADAS-Cog 12 and CDR-sb sub-scales having a correlation of >0.4 within each PCA factor were then grouped according to their association with a specific factor identified by PCA and then labeled as factors 1 through 4 (**Figure 2B**). These composite variables were used as the outcome measures for further analysis by MLM as described above. *Post-hoc* stratification was performed if the interaction terms for treatment, time and baseline AD severity showed a *p*-value ≤ 0.05.

Changes in CSF levels of Aβ38, Aβ40, Aβ42, total tau and P181 tau were calculated by subtracting values of the samples collected at the final visit from the baseline visit for each subject. Given the small sample size for the CSF subset, group comparisons using ANOVA were limited to mild (MMSE ≥ 20) and moderate (MMSE < 20) AD severity categories only. *P*-values ≤ 0.05 were considered significant and all analyses were conducted using SPSS version 24 (IBM, NY).

## Results

### Exploratory Analyses for Subgroup Identification

**Figure 1:**
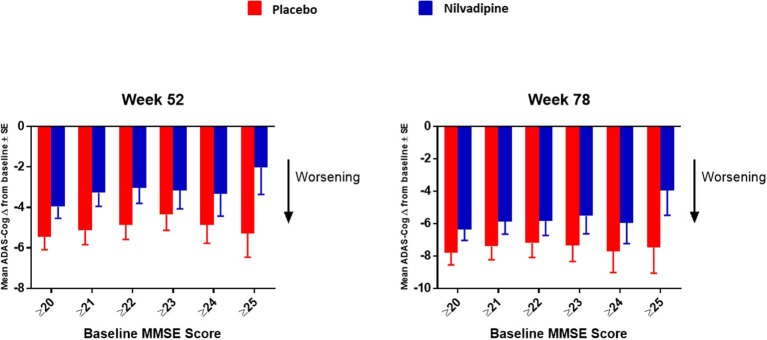
Further stratification of the mild AD group by increasing the increment of MMSE scores by 1 starting from ≥20 up to ≥25. Mean ± SE (for MMSE ≥ 20 *n* = 154 for nilvadipine and *n* = 157 for placebo; ≥21 *n* = 125 for nilvadipine and 136 for placebo; ≥22 *n* = 103 for nilvadipine and *n* = 117 for placebo; ≥23 *n* = 70 for nilvadipine and *n* = 99 for placebo; ≥ 24 *n* = 57 for nilvadipine and *n* = 68 for placebo; ≥25 *n* = 36 for nilvadipine and *n* = 44 for placebo). Mean change from baseline for the total ADAS-Cog 12 scores show the least decline among nilvadipine-treated subjects compared to placebo-treated subjects with MMSE score of ≥25.

**Figure 2:**
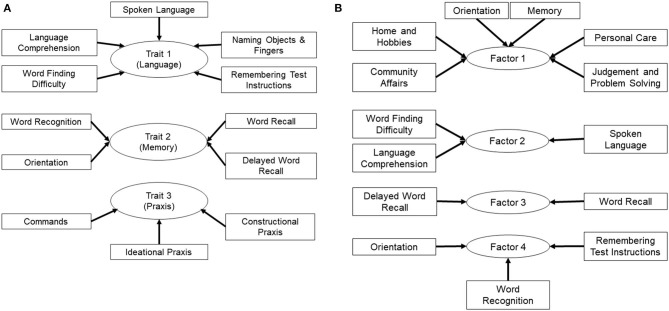
Grouping of ADAS-cog and CDR-sb sub-scales to examine specific cognitive traits **(A)** Sub-scales of the ADAS-cog 12 test were grouped based on traits for memory, language and praxis to account for the topography of tissue loss in AD depending on the stage of disease. **(B)** Sub-scales of the ADAS-cog 12 and the CDR-sb were analyzed by PCA, which resulted in grouping of sub-scales into four factors that explained most of the variance in the dataset. Composite variables were then generated, which included sub-scales identified in each factor by PCA, and were named factors 1 through 4. Note, factor 1 also contains the orientation sub-scale from the ADAS-Cog in addition to the ones from the CDR-sb.

The objectives of these exploratory analyses were to identify a subset of subjects who may have responded differentially to nilvadipine intervention and to facilitate hypothesis development for future studies. The exploratory analyses of the NILVAD trial were restricted to the co-primary outcome measures of ADAS-Cog 12 and CDR-sb. Using a data-driven approach, the mild AD group was further stratified by single point increases in the baseline MMSE scores ranging from 20 to 25 and above. From these results, we generated additional AD subcategories where the AD group with baseline MMSE score > 20 from the NILVAD dataset was further stratified into mild AD (MMSE scores from 20 to 24) and very mild AD (MMSE scores ≥ 25). The moderate AD group (baseline MMSE scores of ≤ 19) was defined as in the original study (18). Demographic characteristics of the AD subgroups stratified by treatment are presented in Table 1. Figure 1 shows ADAS-Cog-12 change from baseline stratified by MMSE scores of the original mild AD group. The nomenclature of mild and very mild AD was adopted in accordance with (28). These analyses also explored the potential impact of nilvadipine treatment on cognitive sub-scales of the ADAS-Cog 12 and CDR-sb tests. The ADAS-Cog 12 sub-scales are: immediate word recall, delayed recall, naming, following commands, constructional praxis, ideational praxis, orientation, word recognition, remembering test directions and instructions, spoken language, comprehension and word finding difficulty in spontaneous speech. The sub-scales of CDR-sb are: memory, orientation, judgment and problem solving, community affairs, home and hobbies and personal care. In addition, ADAS-Cog 12 sub-scales were further grouped into specific traits for memory, language and praxis based on the topography of tissue loss in AD depending on the stage of the disease, as previously suggested by 19. Using this strategy, sub-scales related to each trait were grouped together to generate a composite variable for each trait (Figure 2A). We also calculated a modified AD Composite Score (ADCOMS) using the partial least square (PLS) coefficients previously described elsewhere (20, 29). The ADCOMS is increasingly being used in clinical trials to capture the broad cognitive impairment, particularly in early stage and MCI patient groups. The ADCOMS contains a list of selected items from the ADAS-Cog 12, CDR-sb and the MMSE. In particular, the ADAS-Cog 12 items were: delayed word recall, orientation (for time, place and person), word recognition and word finding difficulty; the CDR-sb items were: personal care, community affairs, home and hobbies, judgment and problem solving, memory and orientation (time, place and person). Normally, for ADCOMS, two additional items are included from the MMSE tests: copying a drawing and orientation for time. However, as we had no follow-up MMSE and as there was no direct drawing equivalent in the ADAS-Cog 12, we could only substitute the orientation item for the missing MMSE orientation item. The numerical equivalency of the derived MMSE score was simply generated by multiplying the ratio of the maximum possible orientation scores for the MMSE and the ADAS-Cog 12 (i.e., 5/8).

**Table 1:** Demographic breakdown of the study population by baseline AD severity.

	**Moderate AD**		**Mild AD**		**Very mild AD**	
	**MMSE ≤ 19**		**MMSE 20-24**		**MMSE ≥ 25**	
	**Nilvadipine**	**Placebo**	**Nilvadipine**	**Placebo**	**Nilvadipine**	**Placebo**
	***N* = 92**	***N* = 94**	***N* = 118**	***N* = 113**	***N* = 36**	***N* = 44**
Age at randomization	71.80 (0.95)	71.79 (0.85)	74.57 (0.77)	73.16 (0.71)	72.38 (1.25)	73.80 (1.16)
Baseline MMSE	16.28 (0.24)	16.23 (0.23)	21.79 (0.13)	22.16 (0.13)	25.56 (0.09)	25.39 (0.08)
Baseline ADAS-Cog	43.04 (1.01)	42.95 (1.03)	30.74 (0.63)	30.96 (0.69)	24.53 (0.95)	25.73 (1.07)
Baseline CDR	7.13 (0.31)	6.83 (0.27)	4.54 (0.17)	4.53 (0.22)	3.14 (0.33)	3.24 (0.28)
Baseline ADCOMS	4.08 (0.11)	4.02 (.11)	2.79 (0.07)	2.84 (0.09)	2.04 (0.14)	2.05 (0.10)
Age left education^*^	14.82 (0.36)	16.05 (0.43)	16.62 (0.36)	16.41 (0.35)	18.61 (0.88)	17.70 (0.65)
Years since AD symptoms	4.62 (0.28)	4.56 (0.27)	4.31 (0.24)	4.36 (0.28)	3.53 (0.27)	3.49 (0.31)
Years since AD diagnosis	2.04 (0.20)	1.80 (0.18)	1.62 (0.14)	1.75 (0.18)	1.33 (0.22)	1.35 (0.22)
Female N (%)	66 (71.7)	57 (60.6)	77 (65.3)	69 (61.1)	17 (47.2)	21 (47.7)
Caucasian N (%)	89 (96.7)	91 (96.8)	115 (97.5)	110 (97.3)	36 (100)	43 (97.7)
APOE4 Carrier^*^ N (%)	32/62 (51.6)	33/64 (51.6)	47/75 (62.7)	48/79 (60.8)	15/24 (62.5)	19/25 (76.0)
Height at Baseline (cm)	162.4 (1.07)	164.1 (0.91)	163.7 (0.87)	164.8 (0.81)	165.3 (1.98)	166.2 (1.43)
Weight at Baseline (kg)	67.2 (1.19)	71.0 (1.49)	66.9 (1.09)	69.5 (1.33)	69.8 (2.31)	68.5 (1.99)
BMI at Baseline	25.5 (0.42)	26.4 (0.50)	25.0 (0.36)	25.6 (0.42)	25.5 (0.65)	24.7 (0.49)

* *APOE genotyping was available only for a subset of individuals and the age the subjects left education was significantly different across MMSE categories. P < 0.05. Education information was not available for 6 subjects and time since AD diagnosis was unavailable for 1 subject. Although the mITT dataset was composed of 498 subjects, 1 subject withdrew consent and therefore data on 497 subjects were available for analysis*.

### Changes in the ADCOMS and the Total ADAS-COG 12 Score in Response to Nilvadipine Treatment Are Modified by Baseline Severity of AD

**Figure 3:**
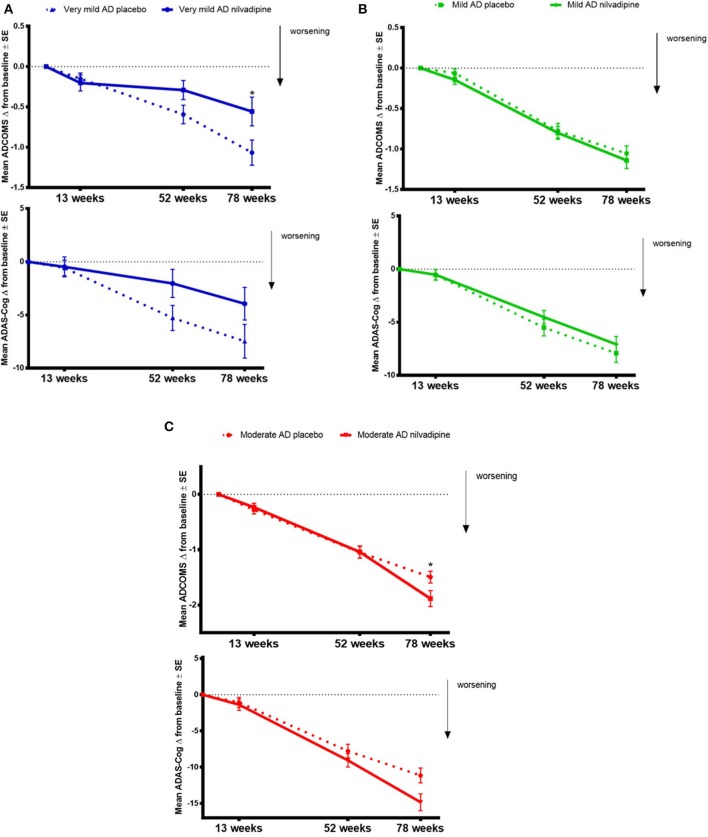
Data on ADCOMS and ADAS-Cog 12 test. Nilvadipine-treated very mild AD subjects show less cognitive decline compared to controls on the ADCOMS and the ADAS-Cog 12 tests. Mean ± SE [*n* = 82 for moderate AD (MMSE ≤ 19) on nilvadipine, *n* = 94 for moderate AD on placebo, *n* = 118 for mild AD (MMSE 20-24) on nilvadipine, *n* = 113 for mild AD on placebo, *n* = 36 for very mild AD (MMSE ≥ 35) on nilvadipine and *n* = 44] for very mild AD on placebo for the change in ADAS-Cog 12 scores. There was a significant effect for the interaction between treatment, time and baseline AD severity as assessed by MMSE scores after correcting for the confounding effects of APOE, gender and education, *p* < 0.05. **(A)** Stratifications show that very mild AD subjects treated with nilvadipine have lower scores on the ADCOMS and the ADAS-Cog 12 compared to placebo after 78 weeks. *post-hoc* analysis stratified by time show a significant treatment effect at 78 weeks for the ADCOMS. **(B)** Mild AD subjects treated with nilvadipine scored similarly to their placebo controls on both the ADCOMS and the ADAS-Cog 12 **(C)**. However, moderate AD subjects treated with nilvadipine scored higher on both the ADCOMS and the ADAS-Cog 12 at 78 weeks compared to those on placebo. *p < 0.05.

We explored whether baseline AD severity modifies the treatment effect of nilvadipine on cognitive decline assessed using the ADCOMS and the ADAS-Cog 12. We observed that the treatment effect of nilvadipine on cognitive decline using the ADCOMS was modified by the baseline severity of AD over the intervention period (*F* = 2.16, *p* = 0.046, Figure 3). *Post-hoc* stratifications showed less cognitive decline on the ADCOMS in very mild AD subjects (*p* = 0.04 at 78 weeks), no change in mild AD subjects (*p* > 0.05) and a greater decline in moderate AD subjects (*p* = 0.03 at 78 weeks) who were treated with nilvadipine compared to their respective placebo treated groups. Similarly, baseline AD severity also modified the treatment response to nilvadipine on cognitive decline detected using the total ADAS-Cog 12 score (*F* = 2.56, *p* = 0.02, Figure 3). In *post-hoc* comparisons, very mild AD subjects had a trend for less cognitive decline observed on the ADAS-Cog 12 test (*p* = 0.06 at 52 weeks) and moderate AD subjects had a greater decline on nilvadipine treatment (*p* = 0.02). There were no differences on the total ADAS-Cog 12 scores between nilvadipine- and placebo-treated individuals from the mild AD group (*p* > 0.05). There were no differences on the CDR-sb total score with respect to the disease severity and treatment (data not shown). Compared to placebo, nilvadipine treated very mild AD individuals performed better on the ADCOMS over 78-weeks irrespective of the ε4 status or gender (Supplementary Figure 1).

### Responses to Nilvadipine on Memory and Language Traits of the ADAS-COG 12 Depend on the Baseline Severity of AD

**Figure 4:**
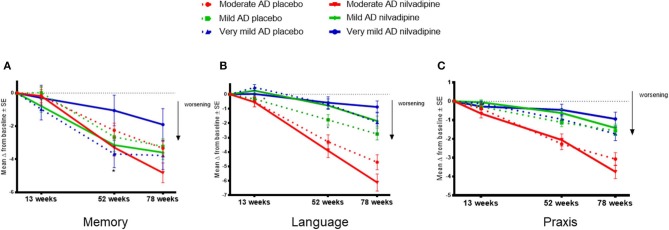
Nilvadipine treatment effects on cognitive traits. Very mild AD subjects show less decline on the memory trait, whereas mild AD subjects show less decline on the language trait, after nilvadipine treatment compared to placebo. Mean ± SE (*n* = 82 for moderate AD on nilvadipine, *n* = 94 for moderate AD on placebo, *n* = 118 for mild AD on nilvadipine, *n* = 113 for mild AD on placebo, *n* = 36 for very mild AD on nilvadipine, *n* = 44 for very mild AD on placebo) for the change in memory, language and praxis traits of grouped ADAS-cog 12 sub-scales. **(A)** There was a significant effect for the interaction between treatment, time and baseline AD severity on the memory trait. *post-hoc* stratifications by time show that very mild AD subjects treated with nilvadipine had significantly less decline on the memory trait compared to their controls. **(B)** There was also a significant interaction between treatment, time and baseline AD severity for the language trait. *post-hoc* stratifications by time show that mild AD subjects treated with nilvadipine had less decline on the language trait compared to the placebo-treated mild AD subjects. **(C)** There was no effect seen for the praxis trait. *p < 0.05.

In order to explore the effects of nilvadipine on cognitive domains that are differentially affected by AD severity, we examined the ADAS-Cog 12 sub-scales grouped as memory, language and praxis traits. These analyses showed that, over the study period, baseline AD severity influenced the treatment response to nilvadipine on the memory trait (*F* = 2.18, *p* = 0.04, Figure 4A). *Post-hoc* stratifications showed that compared to placebo treatment, very mild AD subjects treated with nilvadipine had less decline in the memory trait (*p* = 0.04 at 52 weeks). There were no differences for the memory trait between nilvadipine- and placebo-treated mild AD subjects, while a non-significant decline on the memory trait was noted for moderate AD subjects treated with nilvadipine compared to placebo. Baseline AD severity also influenced the response to nilvadipine on the language trait (*F* = 2.1, *p* = 0.05, Figure 4B) and *post-hoc* stratifications showed less decline in the language trait for the nilvadipine-treated mild AD group only (*p* = 0.03 at 52 weeks). There was no influence of AD severity on treatment effects on the praxis trait (*p* > 0.05, Figure 4C). Similar results were seen with unsupervised PCA of ADAS-Cog 12 and CDR-sb subscales (Supplementary Table 1 and Supplementary Figure 2).

### Nilvadipine Treatment Differentially Modulates CSF Biomarkers Depending on AD Severity

**Figure 5:**
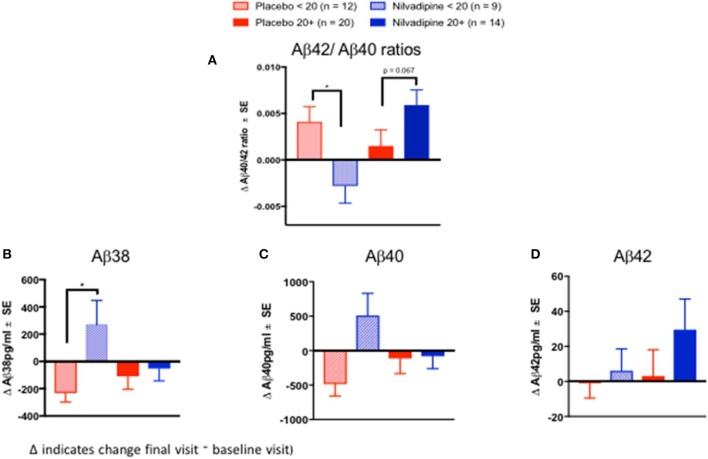
Cerebrospinal fluid Aβ biomarkers. Ratios of CSF Aβ42/Aβ40 increase in nilvadipine-treated mild AD but decrease in moderate AD patients compared to their respective placebo groups. (Mean ± SE *n* = 9 for moderate AD on nilvadipine, *n* = 12 for moderate AD on placebo, *n* = 14 for mild AD on nilvadipine, *n* = 20 for mild AD on placebo). **(A)** Ratios of Aβ42/Aβ40 were higher in mild AD treated with nilvadipine compared to those treated with placebo (*p* = 0.067). There was a significant decrease in Aβ42/Aβ40 in moderate AD treated with nilvadipine compared to placebo. Also in moderate AD subjects, levels of **(B)** Aβ38 and **(C)** Aβ40 were elevated and **(D)** Aβ42 levels were unchanged in moderate AD subjects treated with nilvadipine. Levels of Aβ42 were non-significantly higher in mild AD treated with nilvadipine. *p < 0.05.

**Figure 6:**
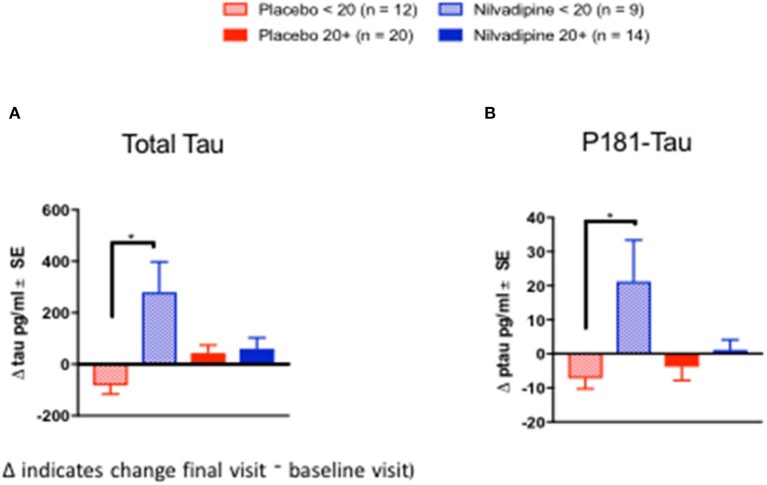
Cerebrospinal fluid tau biomarkers. Total tau and P181Tau levels were increased in nilvadipine-treated moderate AD patients compared to their respective placebo groups. (Mean ± SE *n* = 9 for moderate AD on nilvadipine, *n* = 12 for moderate AD on placebo, *n* = 14 for mild AD on nilvadipine, *n* = 20 for mild AD on placebo). Levels of **(A)** total tau and **(B)** P181 tau were increased in moderate AD subjects treated with nilvadipine. In mild AD subjects, total tau or P181 tau did not differ between nilvadipine-treated and placebo control groups.*p < 0.05.

We examined CSF biomarkers to determine whether treatment response to nilvadipine can be detected using AD biomarkers (see Supplementary Table 2 for baseline demographics of the CSF subcohort stratified by mild and moderate AD severity). Changes in CSF Aβ42/Aβ40 ratios were significantly different across nilvadipine- and placebo-treated mild and moderate AD subjects (*F* = 3.55, *p* = 0.02, Figure 5A). *Post-hoc* analyses showed that CSF Aβ42/Aβ40 ratios showed a significant reduction in moderate AD subjects treated with nilvadipine compared to the placebo group (*p* < 0.05). A trend for an increase in CSF Aβ42/Aβ40 ratios was observed in mild AD cases treated with nilvadipine compared to placebo (*p* = 0.067). Figures 5, 6 show group differences between nilvadipine- and placebo-treated mild and moderate AD subjects for CSF Aβ38 (*F* = 2.98, *p* = 0.04), total tau (*F* = 6.29, *p* < 0.01), and P181 tau (*F* = 4.30, *p* < 0.01). *Post-hoc* analyses showed that in the moderate AD group, nilvadipine treated subjects had significant increases in CSF Aβ38, total tau and P181 tau after nilvadipine treatment (*p* < 0.05). In addition, YKL-40 and neurogranin significantly differed between moderate AD placebo and nilvadipine treated subjects (Supplementary Figure 3).

## Discussion

Many clinical trials in combined populations of mild and moderate AD patients have failed to show overall cognitive benefits. Frequently, the same drugs that have failed in combined mild and moderate populations have suggested cognitive benefits for subjects in mild AD (7–10, 30), when the extent of amyloid and tau pathologies are considerably lower than in moderate AD (31–33). Exploratory analyses of the NILVAD dataset presented here show similar findings where a lower rate of cognitive decline was only seen in the very mild AD group. This effect was detectable primarily on memory related outcome measures that are affected early in the disease process of AD. In addition, the reduced rate of cognitive decline in this group corresponded with a high ratio of CSF Aβ42/Aβ40 after nilvadipine treatment whereas the worsening of cognition in the moderate AD group corresponded with a low ratio of CSF Aβ42/Aβ40. These exploratory studies require further examination to better understand why nilvadipine treatment appears to alter the disease course in very mild AD.

Our current study shows that, compared to their respective placebo groups, the nilvadipine treated very mild AD group experienced less cognitive decline whereas the nilvadipine treated moderate AD group experienced a greater cognitive decline on the ADAS-Cog 12 test and on the ADCOMS. We did not observe an effect of nilvadipine treatment on the changes in CDR-sb. Studies have shown that while the ADAS-Cog 12 test is useful at estimating progression in mild stages of AD, the CDR-sb test is a global impression scale designed for staging of dementia rather than quantifying cognitive change over time. It is therefore possible that the CDR-sb lacks the desired sensitivity to detect subtle cognitive changes due to high test-retest variability for detecting cognitive differences (34, 35). Furthermore, since disease progression in AD differs by the initial stage of the disease, it has also been argued that the ADAS-Cog 12 and CDR-sb tests alone do not have the desired sensitivity to detect subtle changes in cognitive decline that occur in mild AD subjects. Recently, Wang and colleagues developed a composite variable, ADCOMS, which uses subscales from the ADAS-Cog 12 test, the MMSE and the CDR-sb to identify their relative contributions to AD progression (20). This composite outcome includes both cognitive and functional measures. Many clinical trials now incorporate the ADCOMS as it seems to be sensitive at detecting treatment effects in the early stages of AD (36, 37). The use of the ADCOMS (modified to accommodate the absence of MMSE sub-scales) demonstrated reduced cognitive decline in very mild AD subjects treated with nilvadipine.

Subjects in different stages of AD demonstrate differential decline in memory, language and praxis traits. These traits can be mapped to the underlying brain tissue loss in AD in different stages of the disease. Our exploratory analyses of these cognitive traits suggest that benefits of nilvadipine were restricted to the memory trait in very mild AD subjects. In mild AD cases, we observed a reduced decline for the language trait in nilvadipine-treated subjects. There were no effects of nilvadipine on the praxis trait for any of the AD subpopulations. In the moderate AD group, there was no specific domain accounting for the overall decrement in ADAS-Cog 12 with nilvadipine treatment, but rather there were trends for decline in all cognitive domains. Over the 18 months, placebo-treated very mild AD subjects showed a significant decline in memory. This is to be expected, as initially, functional memory is well preserved in very mild AD subjects but then lost rapidly with disease progression. The language trait remained largely preserved in very mild AD subjects treated with placebo but continued to decline further in mild and moderate AD placebo groups. The praxis trait further declined in moderate AD on placebo with minimal decline in both very mild and mild AD on placebo. This is again to be expected as loss of praxis generally occurs after the loss of memory function as AD progresses. Collectively, these data may be another example where use of appropriate cognitive domains relevant to the stage of AD might improve our ability to evaluate treatment effects in AD clinical trials.

Correlative studies of amyloid imaging with CSF Aβ levels show that the decrease in CSF Aβ42 is an early event in AD pathogenesis (33). Recent clinical studies have shown that CSF Aβ42/Aβ40 ratios have a better concordance with amyloid Positron Emission Tomography (PET) imaging for biomarker-based diagnosis of AD than using either Aβ42 or Aβ40 alone (38), and that this ratio is consistently low in AD subjects with high brain amyloid deposition (38, 39). In the present study, in mild AD patients, CSF Aβ42/Aβ40 ratios increased following nilvadipine treatment and this was due to an increase in Aβ42. This would suggest increased clearance of Aβ42 from brain to CSF which is consistent with preclinical studies showing that nilvadipine improves Aβ clearance across biological barriers (13). By contrast, a decline in CSF Aβ42/Aβ40 ratios after nilvadipine treatment in moderate AD subject was due to increases in Aβ40. The decline in CSF Aβ42/Aβ40 corresponded with the worsening of cognition after nilvadipine treatment in this group. As stated above, given that one of the potential mechanisms of action of nilvadipine is to increase Aβ clearance from the brain, the observations of increased CSF Aβ40 and Aβ38 in moderate AD subjects treated with nilvadipine could be interpreted as increased clearance of these shorter Aβ species, rather than Aβ42, from the brain. This may also suggest that removal of Aβ40 and Aβ38 rather than Aβ42 from the brain may be detrimental in the late stages of AD. The proposed clearance of Aβ from the brain is consistent with the results from a NILVAD substudy showing increased cerebral blood flow in the hippocampus after nilvadipine treatment (40), an idea supported by studies showing links between impaired cerebral blood flow corresponding with reduced Aβ clearance from the brain (41). Total tau and P181 tau were increased after nilvadipine treatment in moderate AD subjects. Interestingly, placebo-treated mild and moderate AD subjects showed a decline in total tau and ptau, which is unexpected, but has been previously reported in a longitudinal study of AD subjects (42). Together, biomarker data from this NILVAD trial suggest that cognitive improvement in mild AD subjects after treatment with nilvadipine corresponds to an increase in CSF Aβ42/Aβ40 ratios, whereas worsening of cognition in moderate AD subjects is paralleled by a decrease in CSF Aβ42/Aβ40 ratios and higher total tau, ptau, YKL-40 and neurogranin levels. However, there are some limitations since biomarkers such as amyloid PET imaging data were not available when this clinical trial was designed and initiated. Future studies of nilvadipine in early stage AD subjects should incorporate CSF biomarkers and PET imaging in order to assess the clinical impact on key pathological markers of AD.

## Conclusion

With failures of most AD trials to satisfy efficacy criteria in mixed AD populations, exploratory analyses of existing trial data are justified and necessary to understand lack of efficacy and to identify sub-populations that may have benefited from interventions. The NILVAD trial was designed for the analysis of a mixed mild and moderate AD population and further stratification of the study population into very mild, mild and moderate AD was unplanned and therefore exploratory. As such, these subgroup analyses were underpowered, particularly when considering the confounding effects of gender and APOE. Nevertheless, analyses adjusted for these factors continue to suggest that very mild AD subjects responded positively to nilvadipine on both the ADAS-Cog 12 and the ADCOMS. Furthermore, analyses of the ADAS-Cog 12 sub-scales demonstrate that beneficial effects on memory and language traits were associated with nilvadipine treatment in very mild and mild AD patients, respectively. Together, findings from this clinical study and CSF biomarker analyses suggest a differential response to nilvadipine treatment in AD related to the severity of the disease at treatment initiation. These findings are also consistent with the results of several other experimental AD treatments where only very early stage AD subjects demonstrated benefit, such as Solanezumab (7, 10, 30), aducanumab (8), and LipiDiDiet trials (9). Consequently, the Alzheimer's therapeutic field is increasingly targeting the early stages of AD (43). Finally, possible benefits in the very mild AD group identified by these exploratory analyses warrant further studies of nilvadipine treatment in very mild, prodromal or even preclinical AD patients.

## Data Availability Statement

The datasets generated for this study are available on request to the corresponding author.

## Ethics Statement

The studies involving human participants were reviewed and approved by the IRB of each country where the study approval was received: France: Comite de protection des personnes nord quest III Greece: Scientific Council of Papanikolaou Hospital Thessaloniki Holland: Radboud universitair medisch centrum Concernstaf Kwaliteit en Veiligheid Commissie Mensgebonden Onderzoek Regio Arnhem-Nijmegen (Chair: M.J.J. Prick) Hungary: Medical Research Council Ethics Committee for Clinical Pharmacology (KFEB) (Chair: Dr Fürst Zsuzsarina) Italy: Comitato Etico Istituzioni Ospedaliere Cattoliche (Chair: Dr. Giovanni Zaninetta), Comitato Etico IRCCS MultiMedica (Chair: Prof Emilio Trabucchi), Comitato Etico dell'Aienda Ospedaliera Universitaria S. Martino di Genova (Chair: Dott. Luigi Francesco Meloni) and Comitato Etico Regione Liguria (Chair: Prof. Fulvio Brema), Comitato Etico Fondazione Don Carlo Gnocchi (Chair: Prof Flaminio Cattabeni), Sweden: Regionala etikprövningsnamnden I Göteborg (Chair: Vastra Götalandsregionen) United Kingdom: NRES Committee London – Harrow (Chair: Dr. Jan Downer and Miss Shelly Glaister-Young) Ireland: Tallaght Hospital/St. James's Hospital Joint Research Ethics Committee (Chair: Dr. Peter Lavin) Germany: Ethik-Kommission der Bayerischen Landesarztekammer (Chair: Prof. Dr. med. Joerg Hasford). The patients/participants provided their written informed consent to participate in this study.

## Author Contributions

LA, FC, MM, and BL: conceptualization. LA, GA-G, MT, AB-H, MO, FP, AW, SK, and KB: data curation. LA, DP, and SH: methodology. LA, FC, BL, and MM: project administration and supervision. LA, FC, MT, AB-H, GA-G, DP, MO, FP, AW, SK, SH, KB, BL, and MM: writing—review & editing.

### Conflict of Interest

MM is the Chief Executive Officer of Archer Pharmaceuticals; FC is the Chief Operating Officer of Archer Pharmaceuticals. SH is employed by the Pentara Corporation. KB has served as a consultant or at advisory boards for Alzheon, BioArctic, Biogen, Eli Lilly, Fujirebio Europe, IBL International, Merck, Novartis, Pfizer, and Roche Diagnostics, unrelated to the work presented in this paper. The remaining authors declare that the research was conducted in the absence of any commercial or financial relationships that could be construed as a potential conflict of interest.

## Supplementary Material

Supplemental Figure 1 Evaluation of APOE and gender effects. Changes in total ADAS-Cog 12 stratified by baseline AD severity and APOE ε4 carrier status and gender. Mean ± SE (*n* = 32 for moderate AD on nilvadipine, *n* = 33 for moderate AD on placebo, *n* = 47 for mild AD on nilvadipine, *n* = 48 for mild AD on placebo, *n* = 15 for very mild AD on nilvadipine, *n* = 19 for very mild AD on placebo). **(A)** Moderate non-carrier AD subjects treated with nilvadipine showed decline in the ADAS-Cog 12 and ADCOMS over the 78-week period, whereas mild or moderate ε4 carrier AD subjects treated with nilvadipine scored similarly to their placebo controls. **(B)** Very mild male and female showed less decline compared to their respective controls on ADAS-Cog 12 and ADCOMS over the 78 week period. However, female moderate AD patients declined more compared to male moderate AD patients and its respective placebo group for these outcome measures over the 78 week study period.

Supplemental Figure 2 An Examination of nilvadipine treatment effects using PCA. Nilvadipine-treated mild and very mild AD groups show less decline for PCA Factors 2 and 3, respectively. Mean ± SE (*n* = 82 for moderate AD on nilvadipine, *n* = 94 for moderate AD on placebo, *n* = 118 for mild AD on nilvadipine, *n* = 113 for mild AD on placebo, n = 36 for very mild AD on nilvadipine, *n* = 44 for very mild AD on placebo) for the change in Factors 1, 2, 3, and 4. **(A)** There were no differences between any of the groups for Factor 1. **(B)** A marginally significant interaction between time, treatment and AD severity was observed for Factor 2, *p* = 0.07, and subsequent stratifications show that only mild AD subjects treated with nilvadipine had less decline compared to their placebo controls. **(C)** There was a significant interaction between time, treatment and AD severity for Factor 3, *p* < 0.05. **(D)** There were no significant differences seen between groups for Factor 4.

Supplemental Figure 3 Other CSF biomarkers. Additional CSF biomarker changes in nilvadipine-treated moderate AD patients compared to their respective placebo groups. (Mean ± SE *n* = 9 for moderate AD on nilvadipine, *n* = 12 for moderate AD on placebo, *n* = 14 for mild AD on nilvadipine, *n* = 20 for mild AD on placebo). Levels of **(A)** neurogranin, **(B)** YKL-40 and **(C)** NFL in placebo and nilvadipine treated mild and moderate AD patients. Levels of neurogranin and YKL-40 were elevated in moderate AD subjects treated with nilvadipine compared to placebo. Levels of NFL did not differ in any of the subgroups of mild and moderate AD subjects treated with nilvadipine compared to placebo. ^*^*p* < 0.05.

Supplemental Table 1 Factors identified by PCA from ADAS-Cog 12 and CDR-sb subscales.

Supplemental Table 2 Demographic characteristics of subjects in CSF substudy.
