# Supplementary figures and images for: The Influence of Baseline Alzheimer's Disease Severity on Cognitive Decline and CSF Biomarkers in the NILVAD Trial
Source: Front Neurol. 2020 Mar 6;11:149. doi: 10.3389/fneur.2020.00149 (PMC7067750; doi:10.3389/fneur.2020.00149)

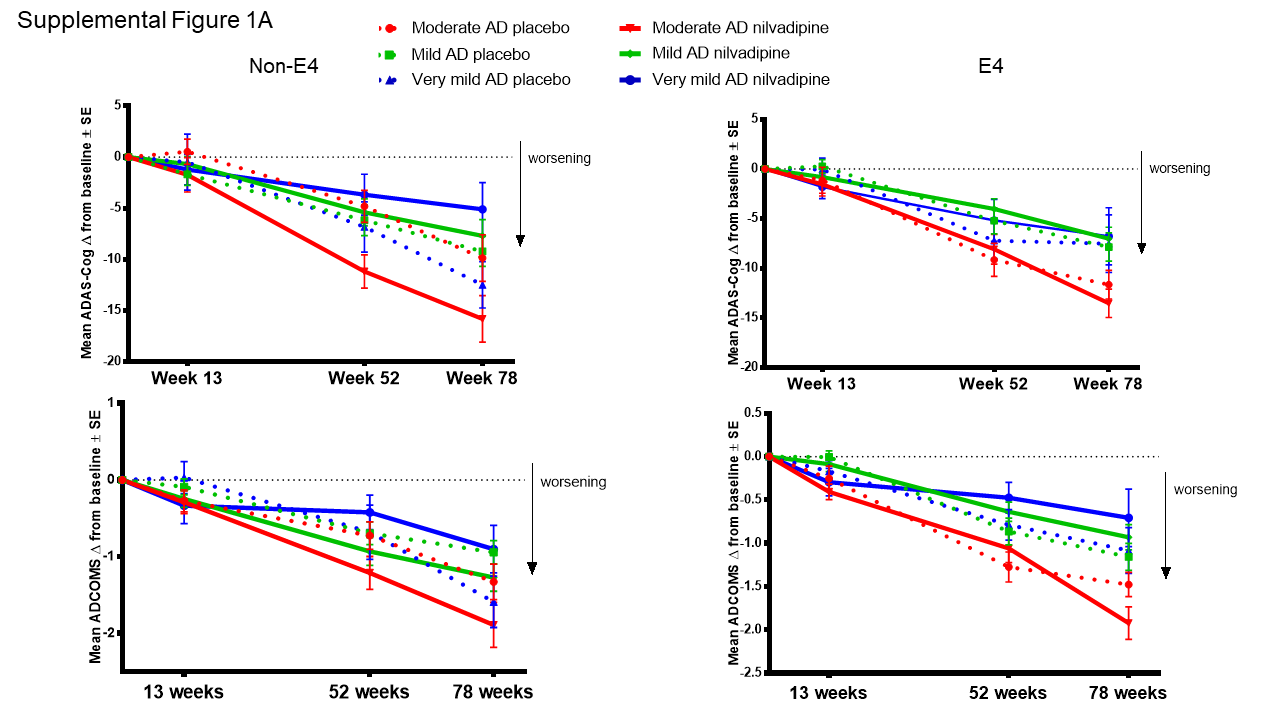

Supplement: Supplementary file 1 [file Image_1.TIF]

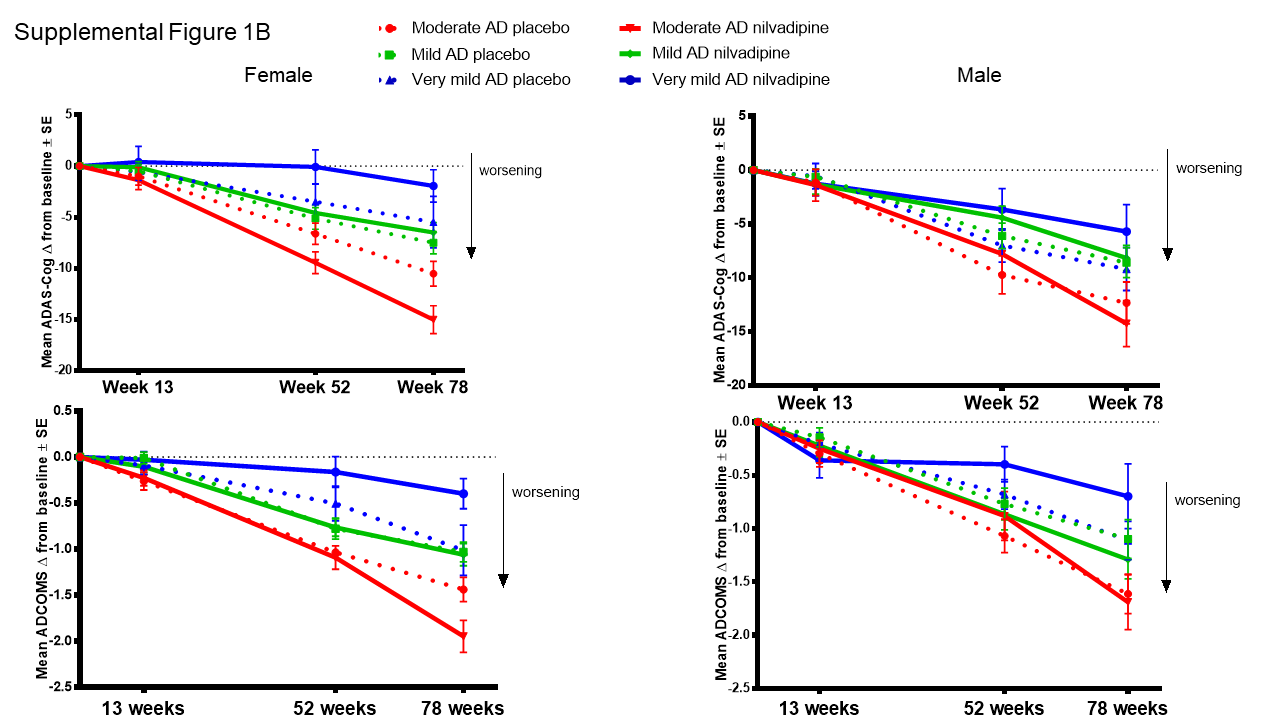

Supplement: Supplemental Figure 1 — Evaluation of APOE and gender effects. Changes in total ADAS-Cog 12 stratified by baseline AD severity and APOE ε4 carrier status and gender. Mean ± SE (n = 32 for moderate AD on nilvadipine, n = 33 for moderate AD on placebo, n = 47 for mild AD on nilvadipine, n = 48 for mild AD on placebo, n = 15 for very mild AD on nilvadipine, n = 19 for very mild AD on placebo). (A) Moderate non-carrier AD subjects treated with nilvadipine showed decline in the ADAS-Cog 12 and ADCOMS over the 78-week period, whereas mild or moderate ε4 carrier AD subjects treated with nilvadipine scored similarly to their placebo controls. (B) Very mild male and female showed less decline compared to their respective controls on ADAS-Cog 12 and ADCOMS over the 78 week period. However, female moderate AD patients declined more compared to male moderate AD patients and its respective placebo group for these outcome measures over the 78 week study period. [file Image_2.TIF]

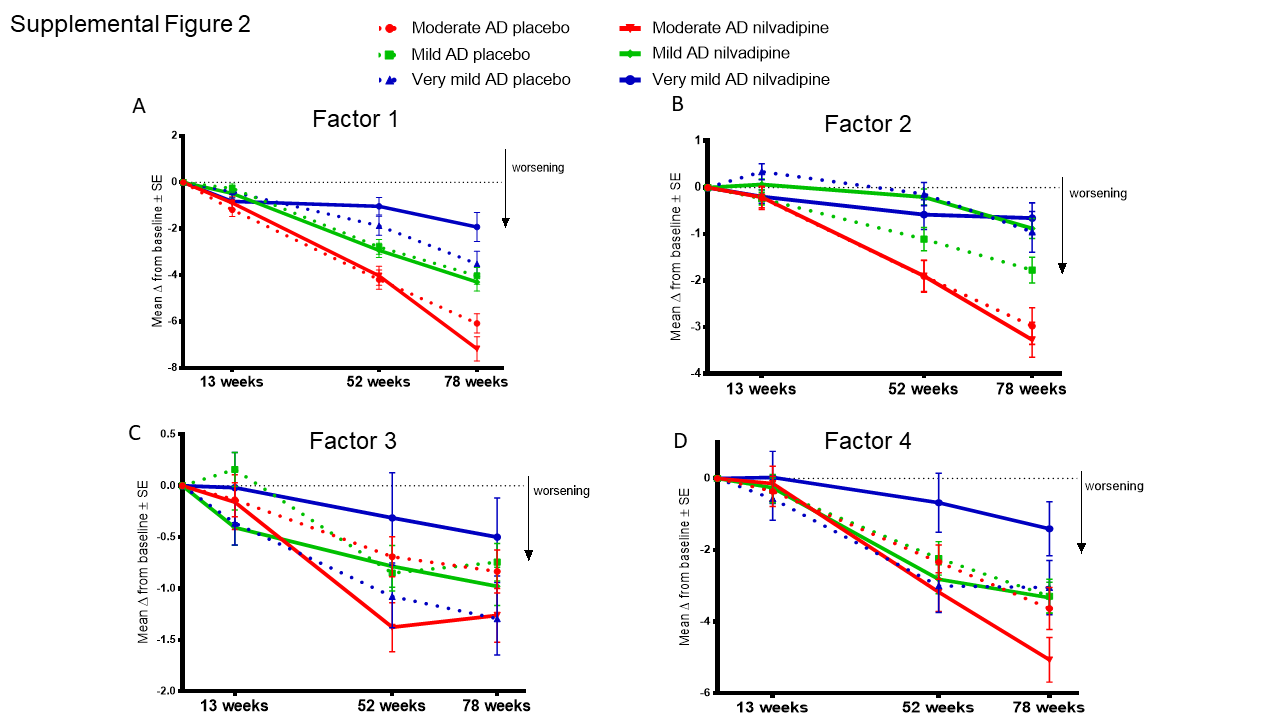

Supplement: Supplemental Figure 2 — An Examination of nilvadipine treatment effects using PCA. Nilvadipine-treated mild and very mild AD groups show less decline for PCA Factors 2 and 3, respectively. Mean ± SE (n = 82 for moderate AD on nilvadipine, n = 94 for moderate AD on placebo, n = 118 for mild AD on nilvadipine, n = 113 for mild AD on placebo, n = 36 for very mild AD on nilvadipine, n = 44 for very mild AD on placebo) for the change in Factors 1, 2, 3, and 4. (A) There were no differences between any of the groups for Factor 1. (B) A marginally significant interaction between time, treatment and AD severity was observed for Factor 2, p = 0.07, and subsequent stratifications show that only mild AD subjects treated with nilvadipine had less decline compared to their placebo controls. (C) There was a significant interaction between time, treatment and AD severity for Factor 3, p < 0.05. (D) There were no significant differences seen between groups for Factor 4. [file Image_3.TIF]

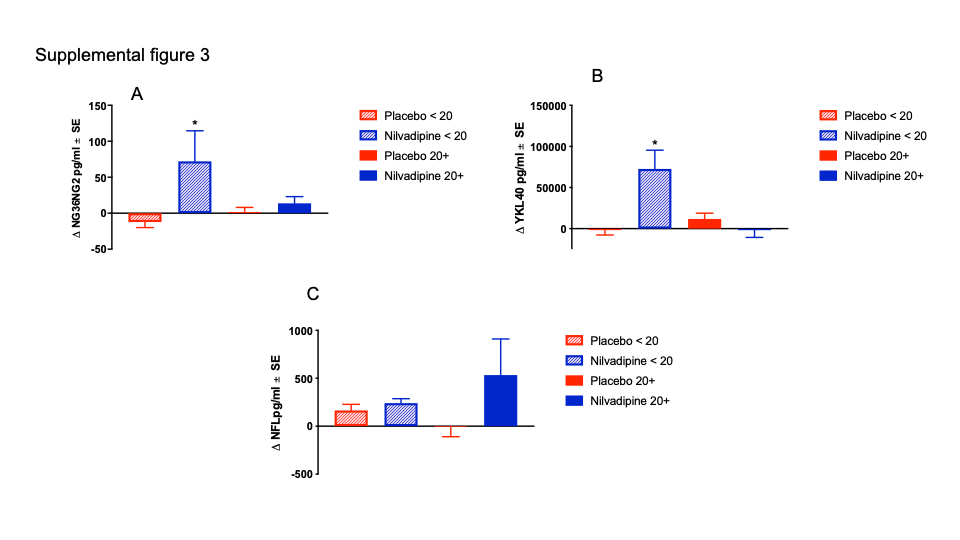

Supplement: Supplemental Figure 3 — Other CSF biomarkers. Additional CSF biomarker changes in nilvadipine-treated moderate AD patients compared to their respective placebo groups. (Mean ± SE n = 9 for moderate AD on nilvadipine, n = 12 for moderate AD on placebo, n = 14 for mild AD on nilvadipine, n = 20 for mild AD on placebo). Levels of (A) neurogranin, (B) YKL-40 and (C) NFL in placebo and nilvadipine treated mild and moderate AD patients. Levels of neurogranin and YKL-40 were elevated in moderate AD subjects treated with nilvadipine compared to placebo. Levels of NFL did not differ in any of the subgroups of mild and moderate AD subjects treated with nilvadipine compared to placebo. *p < 0.05. [file Image_4.TIFF]
